# Fulminating herpes simplex hepatitis

**DOI:** 10.4322/acr.2021.410

**Published:** 2022-12-14

**Authors:** Raheel Ahmed, Kesley Green, Silvio Litovsky, Sameer Al Diffalha

**Affiliations:** 1 University of Alabama Birmingham, Faculty of Medicine, Anatomic Pathology Department, Birmingham, Alabama, USA

**Keywords:** Herpes Simplex Virus, Hepatitis, Crohn’s Disease, Prednisone

## Abstract

Herpes simplex virus (HSV) is a rare cause of acute hepatitis in patients with chronic immunosuppression, including Crohn’s disease. HSV hepatitis has the propensity to cause acute liver failure and death. The presenting signs and symptoms can be nonspecific, thereby causing the diagnosis to go overlooked with inadequate management, leading to a high mortality rate. We report a case of a 31-year-old male on chronic prednisone treatment for Crohn’s disease who unexpectedly died. Subsequently, an autopsy showed HSV hepatitis as the cause of death. Thus, although a rare complication, HSV hepatitis should always be kept in mind as a fatal complication in patients with acute hepatitis and chronic immunosuppression.

## INTRODUCTION

Herpes simplex virus (HSV) hepatitis is a rare complication of HSV infection that has the propensity to cause fulminant liver failure and death. The overall global prevalence of HSV in 2016 was 66.6% for HSV-1 in 0-49-year-old and 13.2% for HSV-2 in 15-49-year-old populations.^1^ Compared to other viral hepatic infections such as Hepatitis B virus and Hepatitis C virus, which are among the top four global infectious disease killers and continue to have increasing mortality rates,^2^ the incidence and prevalence of HSV-1 and 2 causing hepatitis is unknown. HSV hepatitis has been recognized especially in drug-induced immunosuppressed individuals, such as patients with systemic lupus erythematosus, solid organ transplantation, and rectal cancer.^3^^,^ 4 ^,^ 5 It has even been reported in immunocompetent patients.^6^ The prognosis of HSV hepatitis is dismal, with a mortality rate of 80-90%.^7^ Therefore, the early detection and treatment of HSV hepatitis are crucial in preventing rapid clinical deterioration and, ultimately, death.

It is markedly rare to experience HSV hepatitis in the setting of Crohn’s disease. Knösel et al.^8^ studied 11 bacterial and viral infectious agents in the intestinal mucosa of patients who were previously diagnosed with Crohn’s disease. Polymerase chain reaction (PCR) screened for viral and bacterial DNA, only 34% of these screen samples had a positive PCR result. The most prevalent infectious agents were *Stenotrophomonas maltophilia* (17.9%) and Epstein Barr virus (10.7%). There was no positive PCR result for HSV 1 and 2.^8^ There have been; however, a few case reports in the literature of HSV hepatitis in patients with Crohn’s disease.^9^

## CASE REPORT

A 31-year-old male patient with a previous history of Crohn’s disease diagnosed in 2018 and a remote history of cytomegalovirus (CMV) esophagitis was on long-term immunosuppressive therapy, including 60 mg/day of prednisone. His surgical history included a thoracotomy and tibial fracture due to remote trauma, and tooth extraction. He endorsed drinking one to two weekly beers but denied drugs and tobacco use. A week before the current clinical presentation, he was prescribed antibiotics because of dysuria, without improvement. A few days later, he started with chest pain. Subsequently, he was diagnosed with Candida esophagitis and was given a course of fluconazole and prednisone was reduced to 40mg/kg/day. In the following days, the patient’s clinical condition worsened, and he developed nausea, vomiting, and the inability to tolerate oral intake. His symptoms also included shortness of breath, constipation, swollen feet, weakness, and body aches over the last 2 days. On admission, the patient was afebrile, normotensive, and tachycardic. Physical exam was notable for jaundice. The skin was otherwise warm, dry, intact, and free of rashes. His abdomen was soft, mildly distended, and diffusely tender with guarding. He had normal bowel sounds and no organomegaly. No lymphadenopathy was noted.

Initial laboratory findings revealed a white blood cell count of 5.0 x 10^3^/mm^3^ (reference value [RV]: 4,000-11,000 10^3^/mm^3^) with slight neutrophilic predominance (77.7%) (RV: 55-70%), lymphocytopenia (12.1%) (RV: 20-40%), a normal red blood cell count (RBC) of 5.05 x 10^6^/ mm^3^ (RV: 4.35-5.65 x 10^6^/ mm^3^), hemoglobin 14.9 gm/dL (RV: 13.2-16.6 gm/dL), and platelets 47 x 10^3^/ mm^3^ (RV: 150-450 x 10^3^/ mm^3^). Electrolytes were abnormal, revealing potassium of 5.5 mmol/L (RV: 3.6-5.2 mmol/L), calcium 6.6 mg/dL (RV: 8.5-10.2 mg/dL), and an elevated anion gap lactic acidosis of 13.5 mmol/L (RV: 8-12 mmol/L). Kidney function revealed acute kidney injury with an increased creatinine level of 5.47 mg/dL (RV: 0.7-1.3 mg/dL). The alanine aminotransferase (ALT) and aspartate aminotransferase (AST) were elevated at 7291 U/L (RV: 4-36 U/L) and 11811 U/L (RV: 8-33 U/L), respectively. The alkaline phosphatase (ALP) and bilirubin were at 593 IU/L (RV: 44-147 IU/L) and 11.6 mg/dL (RV: 0.1-1.2 mg/dL), respectively. Acetaminophen levels were within the reference range. His international normalized ratio (INR) was 3.2 (RV: 1.0). The viral hepatitis panel was negative for hepatitis A, hepatitis B surface antigen, and core antibody, and within the reference range for Hepatitis C antibody. A chest x-ray was normal. The pelvic computed tomography (CT) was consistent with uncomplicated colitis, which revealed a thickening of the colon wall.

The patient’s clinical status deteriorated within the first 16 hours after admission. The laboratory values revealed a white blood cell count of 3.5 x 10^3^/ mm^3^ and acute anemia with a red blood count (RBC) of 1.86 x 10^6^/ mm^3^ with a severe drop in hemoglobin to 5.6 gm/dL. Platelets decreased to 44 x 10^3^/ mm^3^, and he had worsening lactic acidosis with clinical signs consistent with sepsis. Imaging revealed diffuse bleeding originating from his splenic flexure with possible perforation, and the patient was subsequently taken to the operating room emergently. According to the patient’s operative note, the colon showed spontaneous bleeding from the splenic flexure into the abdominal cavity with evidence of colitis. Furthermore, the perforation of the colon was confirmed by the surgeon during the laparotomy. He received transfused blood products due to profuse bleeding. The bleeding was stopped, and the abdomen was packed with pressure gauze and closed with staples after deciding to leave the pressure gauze in place.

After the surgery, the patient was transferred to the intensive care unit (ICU) for further evaluation. Upon arrival, the patient was intubated and had bilateral central lines, receiving pressors for hypotension. Labs showed abnormal liver enzymes of ALT 1692 U/L, AST 3143 U/L, ALP 156 U/L, marked elevation of serum creatinine, hyperkalemia, hypocalcemia, and lactic acidosis. A massive transfusion protocol was put in place in addition to empiric antibiotics and steroids. Physical exam revealed a markedly distended rigid abdomen, lower extremity mottling, and non-palpable pulses.

The following day, he was retaken to the operating room for possible abdomen compartment syndrome secondary to intra-abdominal bleeding with hemorrhagic shock. Blood products and local coagulation products failed to stop the bleeding. The intraoperative morphological findings suggest of acute liver failure with disseminated intravascular coagulation (DIC). The abdomen was closed, and a negative pressure dressing was placed. His status was switched to do not resuscitate (DNR), and comfort care was implemented. Unfortunately, the patient expired within one day of his initial presentation.

## AUTOPSY FINDINGS

**Figure 1:**
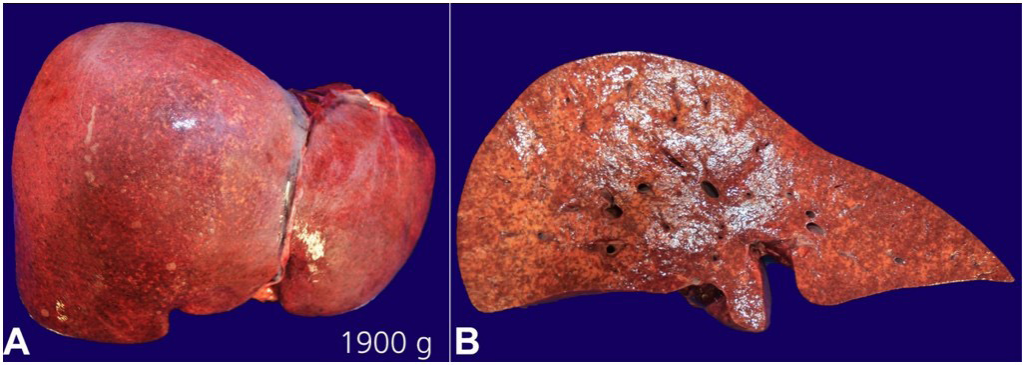
Gross examination of the formalin-fixed liver showing. **A –** hepatomegaly (weight: 1,900 g); **B –** cross section showing areas of necrosis with congestion.

An autopsy was performed 24 hours after the patient’s death. At autopsy, gross findings included hepatomegaly (liver: 1,900 g) (RV: 968-1860 grams) with areas of necrosis and congestion (Figure 1).

**Figure 2:**
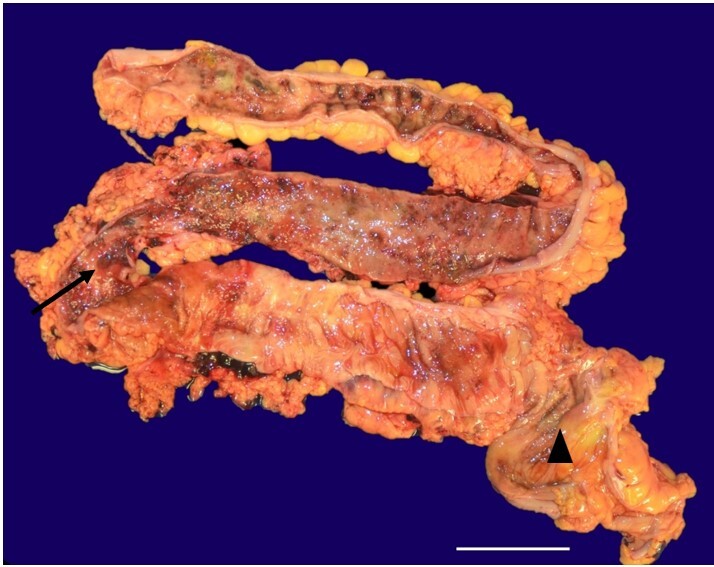
Photomicrography of the ascending, transverse, and descending colon showing multiple ulcers and erosions (arrow) with skip lesions (arrowhead) in a patient with Crohn’s disease (scale bar = 3 cm).

The abdominal cavity contained 500 ml of blood, with the ascending, transverse, and descending colon showing multiple ulcers and erosions with skip lesions (Figure 2). Additional post-mortem findings included bilateral pleural serous effusions and adhesions, focal hemorrhage in the lungs, and pale kidneys.

**Figure 3:**
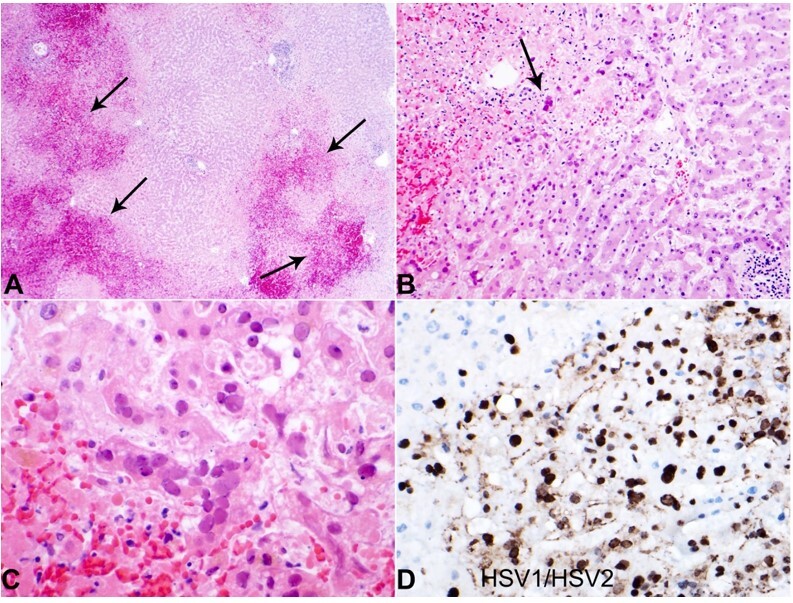
Photography of the liver histology. **A –** Note the low-power areas showing geographic necrosis and hemorrhage (arrows) (H&E 4x); **B –** Low-power section showing viral inclusions (arrow) (H&E 10x); **C –** High power showing HSV inclusions with nuclear molding, margination, and multinucleation (H&E 20x); **D –** HSV1/HSV2 immunohistochemical stain highlighting HSV inclusions within hepatocytes (H&E 20x).

Liver histology revealed the presence of geographic (nonzonal) hemorrhagic necrosis and the classic nuclear cytopathic effect of herpes infection, including margination, multinucleation, and molding (Figure 3A-C). Subsequent immunohistochemical staining with HSV1 and HSV2 was performed, highlighting viral cytopathic effects in hepatocytes, consistent with the herpes simplex virus (Figure 3D).

**Figure 4:**
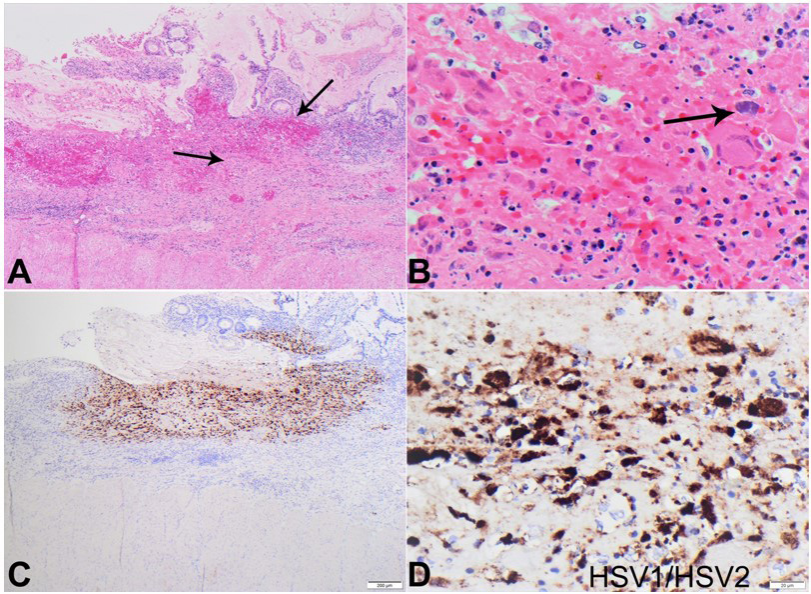
Photography of the colon histology. **A –** Note the low-power areas showing areas of hemorrhage and necrosis in the mucosa of the colon (arrows) (H&E 4x); **B –** Low-power section showing viral inclusions (arrow) (H&E 10x); **C –** Low-power section highlighting HSV inclusions in the colonic mucosa by HSV1/HSV2 immunohistochemical stain (H&E 4x); **D –** High power section highlighting HSV inclusions in the colonic mucosa by HSV1/HSV2 immunohistochemical stain (H&E 20x).

In addition, there was histologic evidence of HSV dissemination to the colon from the sections obtained with associated HSV1/HSV2 positivity with immunohistochemical staining (Figure 4). There was no histologic evidence of active colitis including crypt abscesses, Paneth cell metaplasia, or non-caseating granulomas in the examined sections. The cause of death was disseminated HSV hepatitis involving the colon and liver due to underlying immunosuppression leading to liver failure with severe coagulopathy.

## DISCUSSION

HSV is a prevalent diagnosis globally. It can be seen in a greater number of females (21.7%) than males (11.3%) in the United States in the age group of 15-49 with positive testing for HSV2.^10^ Females in the Americas also have a higher prevalence of testing positive for HSV1.^11^ HSV sepsis can lead to encephalitis, pneumonia, and esophagitis.^12^ It also has a rare manifestation causing fulminant hepatitis in immunocompetent and immunosuppressed patients. It accounts for 1% of acute liver failure cases and has mortality rates reaching 90%.^6^^,^ 12

Diagnosing HSV hepatitis can be difficult due to the nonspecific clinical features. Most HSV infections are asymptomatic or only produce mild nonspecific viral symptoms. Physically, there may be fever, nausea, abdominal pain, or myalgias.^3^ Laboratory findings can include increased transaminases and disseminated intravascular coagulation (DIC). Clinical signs associated with mortality include hypotension, disseminated intravascular coagulation, metabolic acidosis, gastrointestinal bleeding, and bacteremia.^4^

Diagnostic modalities can be challenging to interpret as well. The necrotizing liver lesions on a computed tomography (CT) scan can mimic hepatic abscesses.^13^ Another study notes that CT and magnetic resonance imaging (MRI) showed ring-enhancing hepatic lesions compatible with liver abscesses. However, a subsequent liver biopsy revealed hepatocellular necrosis.^14^ Therefore, the histological correlation may not necessarily coincide with the clinical impression, further complicating the diagnosis of HSV hepatitis. In this case, the liver CT scan showed mild steatosis with no evidence of hepatocellular necrosis. Furthermore, the RT-PCR for HSV was not performed as HSV hepatitis was not initially clinically suspected.

Empiric treatment with acyclovir has improved clinical symptoms of HSV hepatitis and decreased mortality.^3^^,^ 15 ^,^ 16 Beck et al.^17^ reported that early empiric treatment with acyclovir and possible liver transplant increased the survival of patients with fulminant liver failure secondary to HSV. Although acyclovir can reduce the mortality rate in HSV hepatitis, death rates are still significant. Pathologically, HSV hepatitis has two forms, focal and diffuse. According to the publication by Kusne et al.,^18^ twelve patients developed herpes simplex virus (HSV) hepatitis a median of 18 days after solid organ transplantation. All three patients with the diffuse liver disease died. However, three of seven with focal liver involvement survived with antiviral therapy, which suggests that early diagnosis and treatment may be lifesaving. None of these patients had received prophylactic acyclovir. Acyclovir prophylaxis may be able to prevent this disease.^19^ The importance of recognizing high-risk patients and diagnosing HSV hepatitis early in the clinical course has been stressed.^20^ Early empirical antiviral therapy should be considered in immunosuppressed patients with a hepatitis presentation, whether icteric or anicteric.

## CONCLUSION

While Herpes simplex virus is a prominent global diagnosis, herpes simplex virus hepatitis remains a rare complication. It has been noted in both immunocompetent and immunosuppressed patients, such as patients with Crohn’s disease. The presenting symptoms are often vague and nonspecific. The patient often is asymptomatic at presentation or has flu-like symptoms. Many patients present without jaundice or icterus. Diagnostic modalities such as CT and MRI can show nonspecific findings that mimic hepatic abscesses or portal edema. A prompt PCR diagnosis, later followed by empiric treatment with acyclovir, can help improve the patient’s prognosis. Empiric treatment has not yet been established for the treatment of at-risk patients that present with vague symptoms. However, there is evidence that acyclovir can improve the clinical presentation of patients with HSV hepatitis and should be tried to reduce the mortality rate of HSV hepatitis.

## References

[1] James C, Harfouche M, Welton NJ (2020). Herpes simplex virus: global infection prevalence and incidence estimates, 2016. Bull World Health Organ.

[2] Thomas DL (2019). Global elimination of chronic hepatitis. N Engl J Med.

[3] Ahmed A, Granillo A, Burns E (2020). Herpes simplex virus-2 hepatitis: a case report and review of the literature. Case Rep Med.

[4] Kusne S, Schwartz M, Breinig MK (1991). Herpes simplex virus hepatitis after solid organ transplantation in adults. J Infect Dis.

[5] Toomey DP, Dhadda AS, Sanni LA, Cooke JP, Hartley JE (2014). Fatal herpes simplex virus hepatitis following neoadjuvant chemoradiotherapy and anterior resection for rectal cancer. Ann R Coll Surg Engl.

[6] Poley RA, Snowdon JF, Howes DW (2011). Herpes simplex virus hepatitis in an immunocompetent adult: a fatal outcome due to liver failure. Case Rep Crit Care.

[7] Mehta A, Down C, Salama G, Hissong EM, Rosenblatt R, Cantor M (2016). Herpes Simplex Virus Hepatitis in an Immunocompetent Host Resembling Hepatic Pyogenic Abscesses: 1839. Am J Gastroenterol.

[8] Knösel T, Schewe C, Petersen N, Dietel M, Petersen I (2009). Prevalence of infectious pathogens in Crohn’s disease. Pathol Res Pract.

[9] Haag LM, Hofmann J, Kredel LI (2015). Herpes simplex virus sepsis in a young woman with Crohn’s disease. J Crohns Colitis.

[10] Satterwhite CL, Torrone E, Meites E (2013). Sexually transmitted infections among US women and men: prevalence and incidence estimates, 2008. Sex Transm Dis.

[11] Looker KJ, Magaret AS, May MT (2015). Global and regional estimates of prevalent and incident herpes simplex virus type 1 infections in 2012. PLoS One.

[12] Riediger C, Sauer P, Matevossian E, Müller MW, Büchler P, Friess H (2009). Herpes simplex virus sepsis and acute liver failure. Clin Transplant.

[13] Wolfsen HC, Bolen JW, Bowen JL, Fenster LF (1993). Fulminant herpes hepatitis mimicking hepatic abscesses. J Clin Gastroenterol.

[14] Down C, Mehta A, Salama G (2016). Herpes simplex virus hepatitis in an immunocompetent host resembling hepatic pyogenic abscesses. Case Reports Hepatol.

[15] Mortelé KJ, Segatto E, Ros PR (2004). The infected liver: radiologic-pathologic correlation. Radiographics.

[16] Longerich T, Eisenbach C, Penzel R (2005). Recurrent herpes simplex virus hepatitis after liver retransplantation despite acyclovir therapy. Liver Transpl.

[17] Beck J, Haggard H, Membreno F (2020). The first documented case of fulminant liver failure from herpes simplex virus in an immunocompromised patient taking to facitinib. GastroHep.

[18] Kusne S, Schwartz M, Breinig MK (1991). Herpes simplex virus hepatitis after solid organ transplantation in adults. J Infect Dis.

[19] Natu A, Iuppa G, Packer CD (2017). Herpes simplex virus hepatitis: a presentation of multi-institutional cases to promote early diagnosis and management of the disease. Case Reports Hepatol.

[20] Sharma S, Mosunjac M (2004). Herpes simplex hepatitis in adults: a search for muco-cutaneous clues. J Clin Gastroenterol.

